# Molecular targeting of prostate cancer cells by a triple drug combination down-regulates integrin driven adhesion processes, delays cell cycle progression and interferes with the cdk-cyclin axis

**DOI:** 10.1186/1471-2407-11-375

**Published:** 2011-08-25

**Authors:** Steffen Wedel, Lukasz Hudak, Jens-Michael Seibel, Jasmina Makarević, Eva Juengel, Igor Tsaur, Ana Waaga-Gasser, Axel Haferkamp, Roman A Blaheta

**Affiliations:** 1 Department of Urology, Goethe-University; Frankfurt am Main; Germany; 2 Department of Surgery I, Molecular Oncology and Immunology, University of Wuerzburg, Wuerzburg; Germany

## Abstract

**Background:**

Single drug use has not achieved satisfactory results in the treatment of prostate cancer, despite application of increasingly widespread targeted therapeutics. In the present study, the combined impact of the mammalian target of rapamycin (mTOR)-inhibitor RAD001, the dual EGFr and VGEFr tyrosine kinase inhibitor AEE788 and the histone deacetylase (HDAC)-inhibitor valproic acid (VPA) on prostate cancer growth and adhesion in vitro was investigated.

**Methods:**

PC-3, DU-145 and LNCaP cells were treated with RAD001, AEE788 or VPA or with a RAD-AEE-VPA combination. Tumor cell growth, cell cycle progression and cell cycle regulating proteins were then investigated by MTT-assay, flow cytometry and western blotting, respectively. Furthermore, tumor cell adhesion to vascular endothelium or to immobilized extracellular matrix proteins as well as migratory properties of the cells was evaluated, and integrin α and β subtypes were analyzed. Finally, effects of drug treatment on cell signaling pathways were determined.

**Results:**

All drugs, separately applied, reduced tumor cell adhesion, migration and growth. A much stronger anti-cancer effect was evoked by the triple drug combination. Particularly, cdk1, 2 and 4 and cyclin B were reduced, whereas p27 was elevated. In addition, simultaneous application of RAD001, AEE788 and VPA altered the membranous, cytoplasmic and gene expression pattern of various integrin α and β subtypes, reduced integrin-linked kinase (ILK) and deactivated focal adhesion kinase (FAK). Signaling analysis revealed that EGFr and the downstream target Akt, as well as p70S6k was distinctly modified in the presence of the drug combination.

**Conclusions:**

Simultaneous targeting of several key proteins in prostate cancer cells provides an advantage over targeting a single pathway. Since strong anti-tumor properties became evident with respect to cell growth and adhesion dynamics, the triple drug combination might provide progress in the treatment of advanced prostate cancer.

## background

Prostate cancer (PC) is a major medical problem facing the male population. It has become the second most common cause of cancer death in men in the United States [1]. In the western world it is the most common solid tumor in men, followed by lung and colorectal cancer. Although PC is highly curable when diagnosed early, 10 to 15% of patients present with metastases at diagnosis [2-4]. Another 30% develop metastases after initially seemingly curative local treatment fails [5]. Surgical or pharmacological castration is widely accepted as the treatment of choice in advanced PC. However, after a period ranging from 14 to 36 months the tumor becomes hormone refractory. The transition to the hormone refractory stage and metastatic progression pose severe problems in clinical management. Currently, docetaxel chemotherapy has been shown to have a small positive impact on survival, with a median survival gain of less than three months [6,7]. Ultimately, patients succumb as a result of advanced disease.

Over the past decade, several novel drugs have been designed to target specific pathways involved in cancer development and progression. It is assumed that reversal of abnormal cell signaling observed in PC may effectively and specifically slow the aggressive behavior of the disease. This might be particularly true for the phosphatidylinositol 3-kinase (PI3K)/Akt/mammalian target of rapamycin (mTOR) signaling network which critically regulates PC growth and dissemination [8]. There is also evidence that intracellular protein tyrosine kinases which are activated by cell surface growth factor receptors (e.g. epidermal growth factor receptor (EGFr) and vascular endothelial growth factor receptor (VEGFr) control PC growth and survival [9,10]. Finally, since histone deacetylases (HDAC) have been demonstrated to be strongly up-regulated in tumor tissue, HDAC-inhibitors are additionally considered to be promising anti-tumor candidates [11].

Encouraging results have been reported from preclinical studies, and a wide range of molecularly targeted therapy is currently being evaluated in clinical trials. However, because of the diversity of advanced PC and its capacity to adapt to changing conditions, modification of only a single pathway may not ensure long-term effects. Rather, tumor cells might develop resistance to the inhibitor by activating surrogate kinases or downstream components. Consequently, inhibition of multiple pathways may be a promising strategy to avoid adverse effects connected with target redundancy.

The present work was based on the hypothesis that combined interference with VEGFr/EGFr, mTOR and HDAC dependent activation processes might be superior to blocking each pathway separately. The effect of a triple drug combination on PC growth and adhesion properties and the underlying molecular background was evaluated using the PC cell lines PC-3, DU-145 and LNCaP. The antitumor agents employed were the mTOR-inhibitor RAD001, the dual EGFr and VGEFr tyrosine kinase inhibitor AEE788 and the HDAC-inhibitor valproic acid (VPA). AEE788 served as the tyrosine kinase inhibitor of choice due to its bispecific properties. VPA was chosen, since it has been employed in clinical practice for more than 40 years. It has a suitable pharmacokinetic profile and negative side effects are moderate and rare [12].

## Methods

### Cell cultures

Human prostate tumor cell lines PC-3, DU-145 and LNCaP were obtained from DSMZ (Braunschweig, Germany). Normal adult prostatic epithelial PNT-2 cells were purchased from Sigma-Aldrich, München, Germany. Tumor and normal cells were grown and subcultured in RPMI 1640 (Gibco/Invitrogen; Karlsruhe, Germany). The medium contained 10% fetal calf serum (FCS), 2% HEPES-buffer (1 M, pH 7.4), 2% glutamine and 1% penicillin/streptomycin. Subcultures from passages 7-11 were selected for experimental use.

Human endothelial cells (HUVEC) were isolated from human umbilical veins and harvested by enzymatic treatment with chymotrypsin. HUVEC were grown in Medium 199 (M199; Biozol, Munich, Germany), supplemented with 10% FCS, 10% pooled human serum, 20 μg/ml endothelial cell growth factor (Boehringer, Mannheim, Germany), 0.1% heparin, 100 ng/ml gentamycin and 20 mM HEPES-buffer (pH 7.4). Subcultures from passages 2-6 were selected for experimental use.

### Drugs

AEE788 (provided by Novartis Pharma AG, Basel, Switzerland) was dissolved in DMSO as a 10 mM stock solution and stored in aliquots at -20°C. Prior to the experiments, AEE788 was diluted in cell culture medium to 1 μM. RAD001 (provided by Novartis Pharma AG, Basel, Switzerland) was dissolved in DMSO as a 10 mM stock solution and stored in aliquots at -20°C. Prior to the experiments, RAD001 was diluted in cell culture medium to 1 nM. VPA (gift from G. L. Pharma GmbH, Lannach, Austria) was used at a final concentration of 1 mM. Prostate carcinoma cells were treated either with 1 μM AEE788 or 1 nM RAD001 for 24 h or with 1 mM VPA for 3 days, or with all compounds in combination, AEE788+RAD001+VPA. AEE788 and RAD001 were then added for the final 24 h. Controls remained untreated. To exclude toxic effects of the compounds, cell viability was determined by trypan blue (Gibco/Invitrogen). For apoptosis detection the expression of Annexin V/propidium iodide (PI) was evaluated using the Annexin V-FITC Apoptosis Detection kit (BD Pharmingen, Heidelberg, Germany). Tumor cells were washed twice with PBS, and then incubated with 5 μl of Annexin V-FITC and 5 μl of PI in the dark for 15 min at RT. Cells were analyzed on a FACScalibur (BD Biosciences, Heidelberg, Germany). The percentage of apoptotic cells (early and late) in each quadrant was calculated using CellQuest software (BD Biosciences).

### Tumor cell adhesion

To analyze tumor cell adhesion, HUVEC were transferred to 6-well multiplates (Falcon Primaria; BD Biosciences) in complete HUVEC-medium. When confluency was reached, PC-3, DU-145 or LNCaP cells were detached from the culture flasks by accutase treatment (PAA Laboratories, Cölbe, Germany) and 0.5 × 10^6 ^cells were then added to the HUVEC monolayer for 1 h, 2 h or 4 h. Subsequently, non-adherent tumor cells were washed off using warmed (37°C) Medium 199. The remaining cells were fixed with 1% glutaraldehyde. Adherent tumor cells, which appeared translucent with a rounded morphology, were counted in five different fields of a defined size (5 × 0.25 mm^2^) using a phase contrast microscope and the mean cellular adhesion rate was calculated.

### Attachment to extracellular matrix components

6-well plates were coated with collagen G (extracted from calfskin, consisting of 90% collagen type I and 10% collagen type III; Seromed; diluted to 400 μg/ml in PBS), laminin (derived from the Engelbreth-Holm-Swarm mouse tumor; BD Biosciences; diluted to 50 μg/ml in PBS), or fibronectin (derived from human plasma; BD Biosciences; diluted to 50 μg/ml in PBS) overnight. Unspecific cell binding was evaluated by culture plates treated with Poly-D-Lysin (Nunc, Wiesbaden, Germany). Plastic dishes served as the background control. Plates were washed with 1% BSA (bovine serum albumin) in PBS to block nonspecific cell adhesion. Thereafter, 0.5 × 10^6 ^tumor cells were added to each well for 60 min. Subsequently, non-adherent tumor cells were washed off, the remaining adherent cells were fixed with 1% glutaraldehyde and counted microscopically. The mean cellular adhesion rate, defined by adherent cells_coated well _- adherent cells_background_, was calculated from five different observation fields.

### Cell migration and invasion

Serum induced cell migration was examined using 6-well Transwell chambers (Greiner, Frickenhausen, Germany) with 8- μm pores, precoated with collagen (400 μg/ml). 0.5 × 10^6 ^PC-3 or LNCaP cells/ml were incubated with VPA, AEE788, RAD001, or the drug combination. Controls remained untreated. To evaluate cell migration, cells were then placed in the upper chamber for 20 h in serum-free medium. The lower chamber contained 10% serum. After incubation, the upper surface of the Transwell membrane was wiped gently with a cotton swab to remove non-migrating cells. Cells which migrated to the lower surface of the membrane were stained using hematoxylin and counted. Graphical results are shown as % inhibition as compared to the 100% untreated control.

### Measurement of tumor cell growth

Cell proliferation was assessed using the 3-(4,5-dimethylthiazol-2-yl)-2,5-diphenyltetrazolium bromide (MTT) dye reduction assay (Roche Diagnostics, Penzberg, Germany). Treated versus non-treated PC-3, DU-145 or LNCaP cells (100 μl, 1 × 10^4 ^cells/ml) were seeded onto 96-well tissue culture plates. After 24, 48 and 72 h, MTT (0.5 mg/ml) was added for an additional 4 h. Thereafter, cells were lysed in a buffer containing 10% SDS in 0.01 M HCl. The plates were allowed to stand overnight at 37°C, 5% CO_2_. Absorbance at 570 nm was determined for each well using a microplate ELISA reader. Each experiment was done in triplicate. After subtracting background absorbance, results were expressed as mean cell number.

### Cell cycle analysis

PC-3, DU-145 or LNCaP cells were grown to 70% confluency and then treated with AEE788, RAD001 or with VPA or with all compounds in combination (controls remained untreated). Cell cycle analyses were carried out after 24 h. After 24 h tumor cell populations were stained with propidium iodide using a Cycle TEST PLUS DNA Reagent Kit (Becton Dickinson) and then subjected to flow cytometry with a FACScan flow cytometer (Becton Dickinson). 10,000 events were collected from each sample. Data acquisition was carried out using Cell-Quest software and cell cycle distribution calculated using the ModFit software (Becton Dickinson). The number of gated cells in G1, G2/M or S-phase was presented as %.

### Integrin surface expression

PC-3 or LNCaP cells were washed in blocking solution (PBS, 0.5% BSA) and then incubated for 60 min at 4 C with phycoerythrin (PE)-conjugated monoclonal antibodies directed against the following integrin subtypes: Anti-α1 (IgG1; clone SR84), anti-α2 (IgG2a; clone 12F1-H6), anti-α3 (IgG1; clone C3II.1), anti-α4 (IgG1; clone 9F10), anti-α5 (IgG1; clone IIA1), anti-α6 (IgG2a; clone GoH3), anti-β1 (IgG1; clone MAR4), anti-β3 (IgG1; clone VI-PL2) or anti-β4 (IgG2a; clone 439-9B; all: BD Biosciences). Integrin expression of tumor cells was then measured using a FACscan (Becton Dickinson; FL-2H (log) channel histogram analysis; 1 × 10^4 ^cells/scan) and expressed as mean fluorescence units. A mouse IgG1-PE (MOPC-21) or IgG2a-PE (G155-178; all: BD Biosciences) was used as an isotype control.

### Real Time (RT) qPCR

RT qPCR was also done in triplicate. cDNA-synthesis was performed using 3 μg of total RNA per sample according to the manufacturer's protocol by AffinityScript QPCR cDNA Synthesis Kit (Stratagene, Amsterdam, Netherlands). Quantitative gene expression analysis by Real Time PCR was performed by the M × 3005 p (Stratagene, Amsterdam, Netherlands) using SYBR-Green SuperArray (SABioscience Corporation, USA) and SuperArray primer sets: GAPDH (NM_002046.3, Hs.592355), integrin α1 (ITGA1, NM_181501, Hs.644352), integrin α2 (ITGA2, NM_002203, Hs.482077), integrin α3 (ITGA3, NM_002204, Hs.265829), integrin α5 (ITGA5, NM_002205, Hs.505654), integrin α6 (ITGA6, NM_000210, Hs.133397), integrin β1 (ITGB1, NM_002211, Hs.643813), integrin β3 (ITGB3, NM_000212, Hs.218040), integrin β4 (ITGB4, NM_000213, Hs.632226; all: SABioscience Corporation). Calculation of the relative expression of each gene was done by the ΔΔCt method in the analysis program of SABioscience Corporation. The housekeeping gene GAPDH was used for normalisation.

### Western blot analysis

To explore cell cycle regulating proteins as well as the whole cellular integrin level, tumor cell lysates were applied to a 7% polyacrylamide gel and electrophoresed for 90 min at 100 V. The protein was then transferred to nitrocellulose membranes. After blocking with non-fat dry milk for 1 h, the membranes were incubated overnight with monoclonal antibodies directed against cell cycle proteins: Cdk1 (IgG1, clone 1), cdk2 (IgG2a, clone 55), cdk4 (IgG1, clone 97), cyclin B (IgG1, clone 18), cyclin D1 (IgG1, clone G124-326), cyclin E (IgG1, clone HE12), Rb (IgG2a, clone 2), Rb2 (IgG2a, clone 10), p21 (IgG1, clone 2G12), p27 (IgG1, clone 57; all: BD Biosciences). Integrins were analyzed using the monoclonal antibodies listed above. Additionally, integrin-related signaling was explored by anti-integrin-linked kinase (ILK; clone 3), anti-focal adhesion kinase (FAK; clone 77) and anti-phospho-specific FAK (pFAK; pY397; clone 18) antibodies (all: BD Biosciences). HRP-conjugated goat-anti-mouse IgG (Upstate Biotechnology, Lake Placid, NY, USA; dilution 1:5.000) served as the secondary antibody. The membranes were briefly incubated with ECL detection reagent (ECL™, Amersham/GE Healthcare, München, Germany) to visualize the proteins and exposed to an x-ray-film (Hyperfilm™ EC™, Amersham/GE Healthcare). β-actin (1:1.000; Sigma, Taufenkirchen, Germany) served as the internal control.

For control purposes, EGF receptor (EGFr) and mTOR signaling were evaluated. Prostate carcinoma cells were treated with each drug alone or with the triple drug combination as indicated above. Cells were then kept for 2 h in serum-free cell culture medium and subsequently stimulated for 30 min with EGF (100 ng/ml). The following monoclonal antibodies were used: Akt (IgG1, clone 55, dilution 1:500), phospho Akt (pAkt; IgG1, clone 104A282, dilution 1:500), ERK1 (IgG1, clone MK12, dilution 1:5000), ERK2 (IgG2b, clone 33, dilution 1:5000), phospho ERK1/2 (pERK; IgG1, clone 20A, dilution 1:1000), EGFr (IgG1, clone 13/EGFR, dilution 1:500), phospho EGFr (pEGFr; IgG1, clone 74, dilution 1:1000; all: BD Biosciences), p70S6k (IgG, clone 49D7, dilution 1:1000), phospho p70S6k (pp70S6k; IgG, clone 108D2, dilution 1:1000; all: New England Biolabs, Frankfurt, Germany).

### Statistics

All experiments were performed 3-6 times. Statistical significance was investigated by the Wilcoxon-Mann-Whitney-U-test. Differences were considered statistically significant at a p value less than 0.05.

## Results

### Analysis of tumor cell growth and cell cycle progression

**Figure 1:**
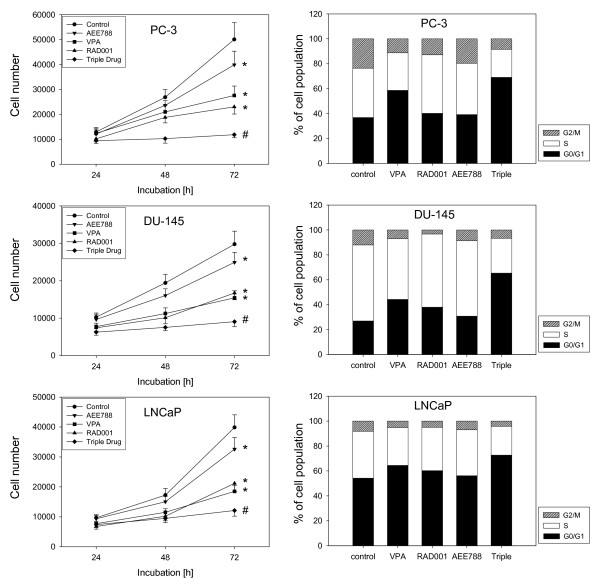
**Cell growth (left) and cell cycle (right) analysis of PC-3, DU-145 or LNCaP cells**. Tumor cells were treated either with 1 μM AEE788, 1 mM VPA or 1 nM RAD001, or with all compounds simultaneously (triple drug), as indicated in the materials section. Controls remained untreated. Cells were counted after 24, 48 and 72 h using the MTT dye reduction assay. One representative experiment of six is shown. Cell cycle analysis was carried out after 24 h. The cell population at each specific checkpoint is expressed as percentage of the total cells analyzed. One representative experiment of three is shown. *indicates significant difference to controls, #indicates significant difference to single drug treatment.

Growth of PC-3, DU-145 or LNCaP cells was inhibited significantly by each drug alone, whereby VPA or RAD001 application was superior to AEE788 treatment (Figure 1). VPA distinctly reduced the amount of G2/M-phase and S-phase cells and strongly enhanced the amount of G0/G1 phase cells. RAD001 particularly diminished the amount of G2/M-phase cells and up-regulated the number of G0/G1 phase cells, which both may account for the observed reduction of tumor growth. With respect to PC-3, the amount of S-phase cells was also slightly elevated, compared to controls, which points to an S-phase arrest as a further mechanism of RAD001.

AEE788 exerted only minor effects on phase shift. The triple drug treatment resulted in a dramatic loss of tumor cell growth, which was more pronounced than growth blockade induced by the single drug regimen. In addition, more cells accumulated in G0/G1 and fewer cells remained in the S-phase, compared to the single drug application. In all experiments, cell growth reduction due to apoptotic events could be excluded as revealed by the Annexin V-FITC assay.

Cell growth studies were also carried out with PNT-2 cells. Cell number of controls moderately increased from 100% to 166 +/- 21% (72 h values). RAD001 did not interfere with this event. VPA (-16.9 +/- 2.2%) and AEE788 (-10.8 +/- 1.9%) slightly but significantly diminished PNT-2 cell number after 72 h, however, not in the magnitude seen with PC-3, DU-145 or LNCaP cells.

### Triple drug treatment alters the expression of cell cycle regulating proteins

**Figure 2:**
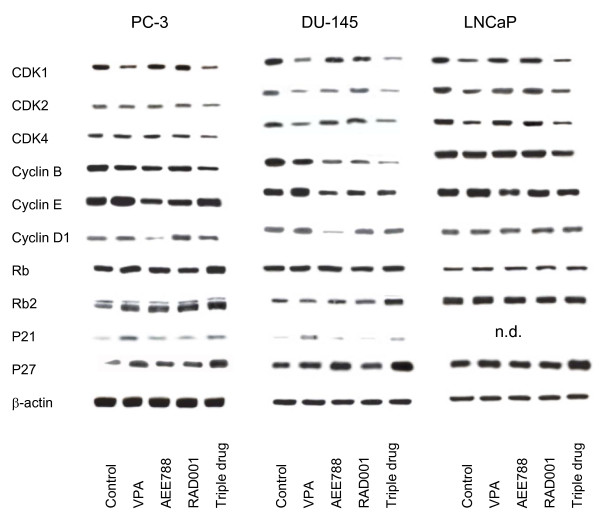
**Western blot analysis of cell cycle proteins, listed in methods**. PC-3, DU-145 or LNCaP cells were treated either with 1 μM AEE788, 1 mM VPA or 1 nM RAD001, or with all compounds simultaneously (triple drug). Controls remained untreated. Cell lysates were then subjected to SDS-PAGE and blotted on the membrane incubated with the respective monoclonal antibodies. β-actin served as the internal control. The figure shows one representative from three separate experiments.

Drug evoked effects on cell cycle proteins depended on both the agent and the cell line used. VPA diminished cdk1 in all prostate cancer cell lines, whereas cdk2 and cdk4 were reduced in DU-145 and LNCaP, but not in PC-3 cells (Figure 2). Cyclin B was reduced in PC-3 and DU-145, but not in LNCaP cells. The opposite was true for cyclin E, which was enhanced in PC-3 and DU-145. p21 was elevated by VPA in PC-3 and DU-145 cells, p27 was highly up-regulated in PC-3 and moderately in DU-145 and LNCaP cells.

AEE788 diminished cyclin B and D1 in PC-3 and DU-145 cells, whereas cyclin E was down-regulated in all cell lines. Elevation of p27 was exclusively evoked in DU-145 cells.

RAD001's effects were particularly seen in blocking cyclin B and E expression. Cyclin D1 was enhanced in PC-3 cells in contrast to the action of AEE788 on this cell line.

Triple drug treatment reduced cdk1, cdk2, cdk4 and cyclin B in all cell lines to a higher extent than did single drug treatment. A combinatorial benefit was also seen with respect to Rb and Rb2. p27 expression was much more elevated in PC-3, DU-145 and LNCaP cells by triple drug use, compared to incubation with each agent alone.

### Down-regulation of tumor cell adhesion and migration by triple drug treatment

**Figure 3:**
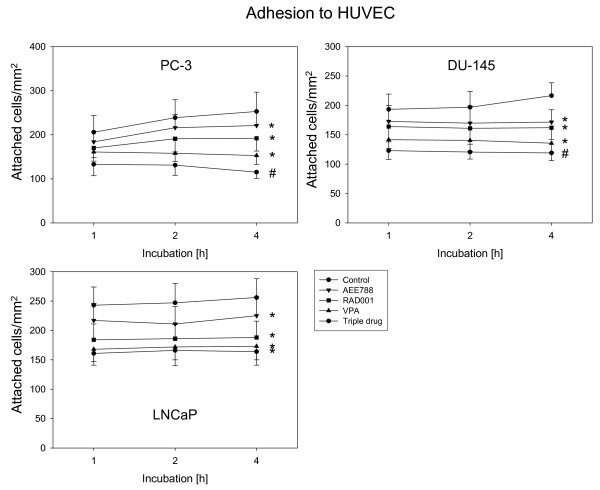
**Adhesion of prostate cancer cells to HUVEC**. PC-3, DU-145 or LNCaP cells were treated with 1 μM AEE788, 1 mM VPA or 1 nM RAD001, applied alone or in combination. Tumor cells were then added at a density of 0.5 × 10^6 ^cells/well to HUVEC monolayers for 1, 2 or 4 h. Non-adherent tumor cells were washed off in each sample, the remaining cells were fixed and counted in five different fields (5 × 0.25 mm^2^) using a phase contrast microscope. Mean values were calculated from five counts. Mean adhesion capacity is depicted as attached cells/mm^2^. One representative of six experiments is shown. *indicates significant difference to controls, #indicates significant difference to single drug treatment.

Subsequent experiments evaluated the impact of the test compounds on prostate cancer cell adhesion. All drugs significantly down-regulated tumor cell attachment to HUVEC, compared to the untreated controls, with VPA being most potent (Figure 3). The combined use of three compounds was superior to single drug application in down-regulating tumor cell attachment with PC-3 and DU-145 but not with LNCaP cells. VPA did not influence PNT-2-HUVEC interaction, whereas AEE788 and RAD001 slightly diminished this process by 23.6 +/- 4.9 or 20.6 +/- 4.7%, respectively (2 h values each). No beneficial effect was seen in presence of the triple drug regimen, compared to single drug treatment.

**Figure 4:**
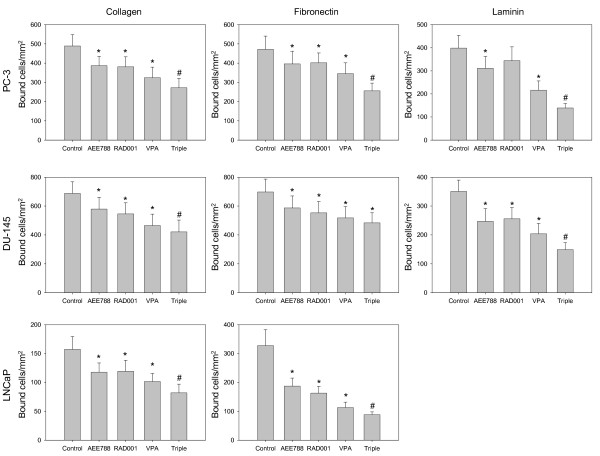
**Adhesion of prostate cancer cells to extracellular matrix proteins**. PC-3, DU-145 or LNCaP cells were treated with 1 μM AEE788, 1 mM VPA or 1 nM RAD001, or with the drug combination (triple). Non-treated cells served as the controls. Cells were then added to immobilized collagen, laminin, or fibronectin at a density of 0.5 × 10^6 ^cells/well for 60 min. Plastic dishes were used to evaluate unspecific binding (background control). Non-adherent tumor cells were washed off in each sample, the remaining cells were fixed and counted in five different fields (5 × 0.25 mm^2^) using a phase contrast microscope. Mean values were calculated from the five counts. Specific adhesion capacity (background adhesion on plastic surface was subtracted from adhesion to matrix proteins) is depicted as bound cells/mm^2^. One representative of six experiments is shown. *indicates significant difference to controls, #indicates significant difference to single drug treatment.

The influence of single versus triple drug treatment on tumor cell binding to extracellular matrix proteins is shown in Figure 4. Binding to immobilized collagen, fibronectin or laminin (PC-3, DU-145) or to immobilized collagen or fibronectin (LNCaP) was strongly blocked by VPA, RAD001 or AEE788. Since untreated LNCaP cells only marginally attached to laminin, drug induced effects on LNCaP-laminin interaction were not analyzed. No drug effects were seen on prostate carcinoma cell lines grown on Poly-D-Lysin coated dishes (data not shown). The triple drug regimen further diminished the amount of attached cells in all assays except the DU145-fibronectin experiment. Binding of PNT-2 cells to collagen revealed no differences between controls and drug treated cells (data not shown).

**Figure 5:**
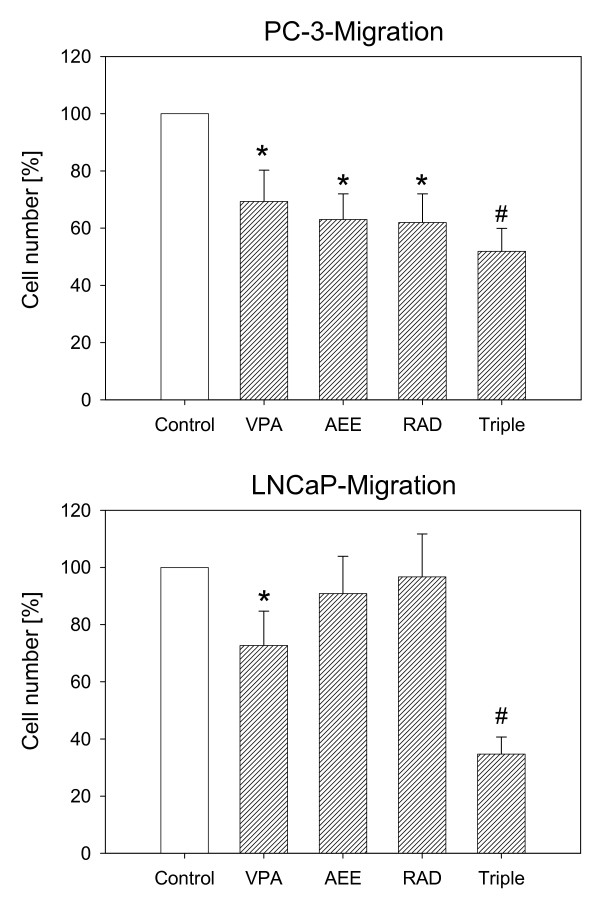
**Analysis of tumor cell migration by the Transwell chamber assay**. To evaluate the migratory potential, drug treated versus non-treated (control) tumor cells were seeded in the upper chamber and 10% serum as the chemoattractant was placed in the lower well. The upper chamber has been coated with collagen. Cells which migrated to the lower surface of the membrane were stained using hematoxylin and counted. Controls were set to 100%. * = significant different to controls. # = significant different to single drug treatment.

Since distinct adhesion differences were seen between LNCaP and DU-145/PC-3 but not between DU-145 and PC-3 cells, subsequent migration experiments were concentrated on PC-3 and LNCaP. In doing so, VPA diminished migration properties of PC-3 and LNCaP cells. AEE788 and RAD001 also acted on PC-3 but not of LNCaP cells (Figure 5). PC-3 and LNCaP migration was further reduced when the three drugs were applied simultaneously.

### Drug treatment alters integrin α and β subtype expression

**Figure 6:**
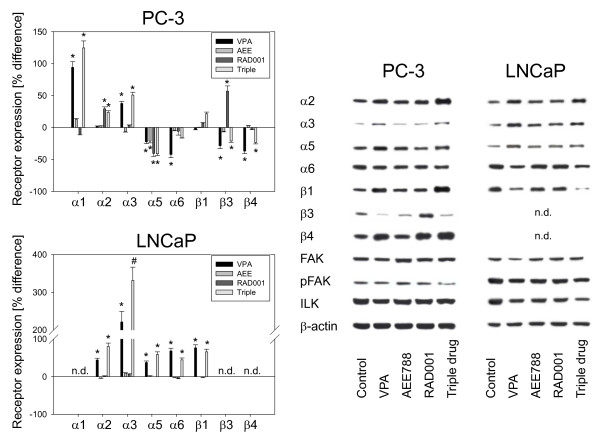
**Analysis of integrin surface expression (left) and intracellular integrin protein level (right)**. PC-3 or LNCaP cells were treated either with 1 μM AEE788, 1 mM VPA or 1 nM RAD001, or with all compounds simultaneously (triple). Non-treated cells served as the controls. To explore integrin surface expression, cells were washed in blocking solution and stained with specific monoclonal antibodies as listed in materials and methods. A mouse IgG1-PE or IgG2a-PE was used as the isotype control. Fluorescence was analysed using a FACScan flow cytometer, and a histogram plot was generated to show PE-fluorescence. The mean fluorescence units are given in percentage difference to the controls. One of three independent experiments is shown here. *indicates significant difference to controls, #indicates significant difference to single drug treatment. To carry out western blotting, cell lysates were subjected to SDS-PAGE and blotted on the membrane incubated with the respective monoclonal antibodies (including anti-ILK, anti-FAK and anti-pFAK). β-actin served as the internal control. The figure shows one representative from three separate experiments.

In ongoing studies, integrin subtype expression was explored in PC-3 and LNCaP cells. Figure 6 (left) depicts the percentage change of integrin surface level induced by single or tripled drug treatment. VPA enhanced α1 and α3 and diminished the α5, α6, β3 and β4 expression level on PC-3 cells. The α4 integrin subtype was not detected on the surface of untreated PC-3 cells (data not shown). Differently from PC-3, VPA induced α2, α3, α5, α6 and β1 up-regulation on LNCaP cells. LNCaP control cells were negative for α1, α4, β3 and β4 integrins. In contrast to VPA, RAD001 elevated α2 and β3 and diminished a5 on PC-3, and enhanced α3 on LNCaP cells. AEE788 exclusively reduced the α5 integrin subtype on PC-3 and up-regulated α3 on LNCaP cells. When tumor cells were exposed to the triple drug regimen, α1 surface expression further increased on PC-3 cells, compared to VPA single drug use, and additive effects were evoked on α3 expression on LNCaP cells.

Western blotting demonstrated enhanced α2, α3, α5, β1 and β4 protein expression accompanied by a diminished α6, β3 and ILK protein level in PC-3 cells when exposed to VPA. VPA also induced α2, α3, α5 elevation and α6 reduction in LNCaP cells. However, the β1 integrin was down-regulated by VPA in this cell line (Figure 6, right). VPA also triggered the loss of ILK and FAK (total and activated). RAD001 enhanced α2, β3 and β4 integrins and reduced both the α5 integrin and ILK in PC-3 cells. It triggered α3 and α5 elevation and simultaneously evoked down-regulation of ILK and pFAK in LNCaP cells. AEE788 diminished β3 in PC-3 cells. Concerning LNCaP cells, the α3 integrin portion was up-regulated, whereas ILK and pFAK were reduced by this compound.

**Figure 7:**
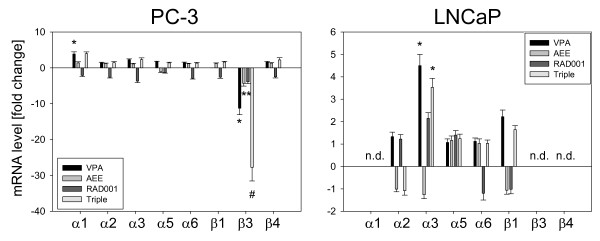
**Analysis of integrin gene expression**. PC-3 or LNCaP cells were treated with 1 μM AEE788, 1 mM VPA or 1 nM RAD001, or with all compounds simultaneously (triple). Non-treated cells served as the controls. Primer sets used for evaluation are listed in materials and methods. Calculation of the relative expression of each gene was done by the ΔΔCt method in the analysis program of SABioscience Corporation. The housekeeping gene GAPDH was used for normalisation. One representative from three separate experiments is shown. *indicates significant difference to controls, #indicates significant difference to single drug treatment.

Analysis of integrin coding genes revealed that VPA considerably reduced the β3 coding mRNA in PC-3 cells (Figure 7). The same effect, although to a lesser extent, was seen when AEE788 or RAD001 was used. An additive action was evoked by the triple drug combination. In contrast, only VPA acted on LNCaP cells by elevating α3 integrin mRNA, and no additive effects were induced by the triple drug protocol.

### Analysis of intracellular signaling

**Figure 8:**
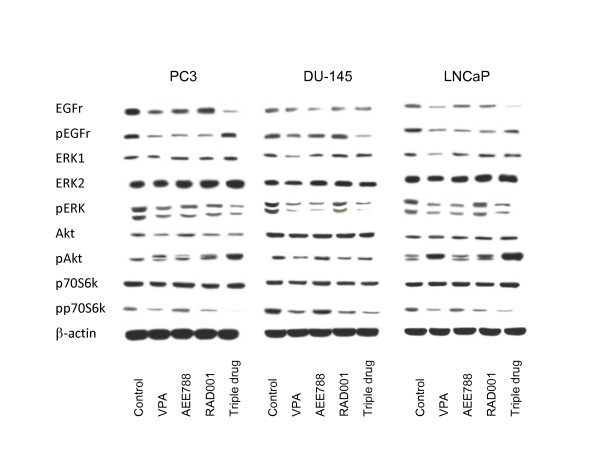
**Western blot analysis of cell signaling proteins, listed in methods**. PC-3, DU-145 or LNCaP cells were treated either with 1 μM AEE788, 1 mM VPA or 1 nM RAD001, or with the triple drug combination (triple drug). Controls remained untreated. Before evaluation, cells were kept for 2 h in serum-free cell culture medium and subsequently stimulated for 30 min with EGF (100 ng/ml). Cell lysates were then subjected to SDS-PAGE and blotted on the membrane incubated with the respective monoclonal antibodies. β-actin served as the internal control. The figure shows one representative from three separate experiments.

The interference of RAD001, AEE788 or VPA with intracellular signaling (Figure 8) was investigated. VPA diminished EGFr (total and activated), pERK and phosphorylated p70S6k in all cell lines. Analysis of pAkt revealed conflicting results, since this protein was distinctly reduced in DU-145, strongly enhanced in LNCaP, whereas a protein double band appeared in PC-3 cells. Both, pEGFr and pERK were down-regulated in all tumor cells following AEE788 exposure, but pp70S6k expression was similar between treated and untreated cells. The latter was also true with respect to pAkt. RAD001 reduced pEGFr in PC-3 and LNCaP and pERK in PC-3 and DU-145 cells. RAD001 also down-regulated pp70s6 k in all explored cell lines.

Triple drug treatment provided combinatorial benefit with respect to EGFr (PC-3, LNCaP), pEGFr (DU-145), pERK (all cell lines) and pp70S6k (all cell lines) loss. Furthermore, the amount of pAkt proteins was greatly elevated in PC-3 and LNCaP cells, exceeding the pAkt levels evoked by single drug use. pEGFr down-regulation induced by single drug treatment in PC-3 and LNCaP cells was reverted by the triple drug application.

## Discussion

The combined inhibition of EGFr/VEGFr and mTOR related pathways, coupled with HDAC deactivation, profoundly blocked PC growth and adhesion. The blocking effect was similar in all employed cancer cell lines and more extensive, compared to the single drug regimen. This is important, since each compound interferes with the tumor's molecular machinery differently. Cdk2 and cdk4 were diminished by VPA in DU-145 and LNCaP but not in PC-3 cells. Cyclin E was elevated by VPA but reduced by AEE788. RAD001 profoundly altered cyclin B in DU-145 but not in PC-3 and LNCaP cells. Several investigators have recently demonstrated that a tumor cell's response to a particular drug depends on receptor and protein configuration, which is characteristic in the different PC cell lines [13,14]. It has been shown that the PC phenotype determines its sensitivity towards treatment with a tyrosine kinase inhibitor [15], mTOR [16] or HDAC-inhibitor [17].

The variable response of the cell lines to a single drug treatment is not foreseeable, due to the PC's heterogeneous nature, resulting in different malignant maturation pathways and protein profiling. Analysis of mTOR in PC patients revealed distinct heterogeneity in the study cohort [18]. The same was true with respect to EGFr and VEGF expression [19], and to the HDAC level [20]. Given the molecular specificity of each targeted compound, it is unrealistic to expect similar biochemical reactions in every PC cell line. The data presented here demonstrate that the triple drug combination circumvents this problem by exerting anti cancer properties in different tumor cell types according to the particular molecular profile. From a clinical viewpoint, simultaneous use of a set of drugs with complementary pharmacological characteristics may enhance the total percentage of responders, as well as the elimination rate of tumor clones in each individual patient. The VPA-RAD001-AEE788 drug combination diminished cdk1, cdk2, cdk4 and cyclin B in PC-3, DU-145 and LNCaP cells to a similar extent, although each compound modified these proteins differently when given separately. In a TRAMP mouse model, it has been shown that PC growth and progression is regulated by these proteins and that blocking cdk2, cdk4 and cyclin B expression results in suppression of cell cycle progression and cell proliferation [21]. There is also evidence that therapeutic elevation of Rb2 and p27 contributes to PC prevention [22], and indeed, Rb2 (PC-3, DU-145) and p27 up-regulation was observed when the triple drug combination was applied. The role of p21 is difficult to interpret, since it was only marginally expressed in PC-3 and DU-145 cells and slightly enhanced by the triple drug protocol. Enhancement of p21 has been attributed to growth delay and apoptosis induction [23,24], although reduction of p21 did not hinder this process [25]. Therefore, it may be assumed that p21 plays a minor role in VPA-RAD001-AEE788 evoked cell growth blockade.

A noteworthy phenomenon was seen with cyclin E, becoming elevated by VPA but diminished by AEE788. Controversial data has been published relevant to this phenomenon. HDAC-inhibition led to tumor growth arrest, accompanied by increased levels of cyclin E in leukemia and lung cancer cells [26,27], decreased cyclin E levels in breast cancer [28], whereas cyclin E was not changed in bladder cancer [29]. Information about AEE788 is sparse. AEE788 reduced cyclin E in one (of three) kidney tumor cells [30], which was also inhibited by the dual EGFr and VEGFr inhibitor ZD6474 in breast tumors [31]. Down-regulation of cyclin E also takes place in several tumor types when the tyrosine kinase inhibitors sorafenib or sunitinib was applied [32-34]. Considering that cyclin D1 was simultaneously diminished by AEE788 (in PC-3 and DU-145 cells), we assume that cyclin reduction represents a specific mechanism of this compound. In contrast, VPA's activity on cyclin E may vary with the tumor type. Whether the VPA triggered cyclin E increase in PC contributes to a loss of proliferative capacity, reflects a negative feedback loop or an unspecific phenomenon warrants further evaluation.

Interestingly, moderate growth blocking effects of VPA and AEE788 were also induced on normal prostatic epithelial PNT-2 cells. When interpreting these data, it should be considered that PNT-2 cell lines have been immortalized by introducing the SV40 large T antigen. This procedure significantly alters the physiology of the cells with the consequence that the "normal" cells acquire tumor-specific characteristics [35,36]. Indeed, PNT-2 demonstrated a significant proliferative activity in the MTT-assay, contrasting the behavior of physiologically intact prostate cells. Since the drugs applied act on cell cycle progression, it is not surprising to see moderate anti-proliferative action also on this cell type.

Beside cell growth reduction, the VPA-RAD001-AEE788 combination interfered with processes related to tumor invasion. This is highly relevant, because metastatic spread is the major obstacle in treating PC. Alterations of the integrin adhesion receptors caused by the agents did not reveal a straightforward pattern. Based on PC-3 cells, α2 and β1 integrins were elevated, α6 and β3 integrins reduced, while β4 integrins were diminished on the cell membrane, but the total β4 integrin level (including the cytoplasmic content) was enhanced.

It has recently been demonstrated that blocking β3 or β4 integrin membrane presentation significantly lowers PC cell attachment to endothelium and extracellular matrix [13]. Therefore, prevention of β3 and β4 integrin driven cell-cell or cell-matrix communication might be one mechanism accounting for how the drug combination modulates invasive processes. A positive correlation between β3 or β4 integrin expression and PC metastasis has already been reported [37-39]. A different background should be considered when interpreting β1 (as well as α2) integrin expression. Obviously, the β1 integrin does not exclusively serve as a mechanistic binding receptor but rather transduces signals that inhibit the invasive behavior of epithelial cells [40]. Possibly, the elevation of integrin β1 reported in this investigation may cause the neoplastic phenotype to revert to a less invasive phenotype as has previously been reported [41]. Blocking an antibody to β1 integrin did not impair PC migration in vitro [39], which is in good accordance with this hypothesis. An interesting aspect has been proposed by Goel et al. who discovered that integrin β1 prevents PC cancer progression by up-regulating the secretion of angiogenesis blocking factors [42]. Therefore, use of VPA-RAD001-AEE788 may not only be beneficial in inhibiting tumor growth and invasion but also for counteracting processes related to angiogenesis. Overall, the role of integrins is complex. Beside surface expression, integrin clustering, spatiotemporal dynamics of integrin internalization and recycling determine whether a tumor cell becomes motile or not [43]. It should also be considered that drugs with a chemical structure slightly different from those used in the present investigation may not necessarily induce the same response.

A strong de-activation of p70S6k was induced in all cell lines by the triple drug regimen, which may explain how the drugs diminished PC growth in the present investigation. In fact, attenuation of p70S6k was reported to suppress proliferation of aggressive PC and to trigger autophagic death in vitro [44,45]. Paradoxically, phosphorylation of Akt increased in PC-3 and LNCaP cells following drug exposure. A similar phenomenon has been observed by Sun and coworkers who discovered that suppression of p70S6k activity was paralleled by elevation of Akt activity [46]. The clinical relevance is not clear. Analysis of peripheral blood mononuclear cells in patients treated with RAD001 for relapsed or refractory hematologic malignancies revealed a decreased phosphorylation of both p70S6k and pAkt [47]. The latter report corresponds to the effects of the triple drug combination on DU-145 cells in our assay which were also paralleled by down-regulation of p70S6k and pAkt (in contrast to PC-3 and LNCaP). At this point, it is not possible to determine which of the pAkt modifications reflect the desired pharmacological mode of action and which may be correlated to a negative feedback loop.

## Conclusions

Simultaneous targeting of three intracellular mechanisms profoundly blocks PC cell adhesion and growth in vitro. This strategy may provide a clinical benefit in PC patients. No study data are available so far in this matter. However, there is no doubt that pathway crosstalk should be considered when designing an optimum treatment protocol [48]. Analyses of human PC tissue microarrays demonstrated that Akt/mTOR and growth factor related ERK/MAPK signaling are often coordinately deregulated during PC progression [49]. Carracedo et al. reported that tumor cell biopsies obtained from patients treated in the neoadjuvant setting with RAD001 displayed activation of ERK [50]. The authors concluded that combined interruption of both pathways may significantly increase the efficacy of clinically relevant mTOR inhibitors. When discussing the future role of combined tumor targeting, additional integration of cytotoxic agents should also be considered to improve the current standard of care for men with PC. In fact, combination treatment with docetaxel and several different targeted drugs has shown potential for increased efficacy and good tolerability compared with docetaxel alone [51]. Further investigation including animal studies is now warranted to validate our findings.

## Competing interests

The authors declare that they have no competing interests.

## Authors' contributions

LH and JMS performed all major experimental work of the study. EJ and IT carried out western blotting and cell cycle analysis. JM focused on PNT-2 cells, RAB contributed to the design and coordination of the study and drafted the manuscript. SW drafted the manuscript and performed the data analysis. AWG participated in the conception of the study and data interpretation. AH was involved in the overall design of the study and helped to draft and revised the manuscript. All authors have read and approved the final manuscript.

## Pre-publication history

The pre-publication history for this paper can be accessed here:

http://www.biomedcentral.com/1471-2407/11/375/prepub
